# Potent VEGFR-2 inhibitors for resistant breast cancer: a comprehensive 3D-QSAR, ADMET, molecular docking and MMPBSA calculation on triazolopyrazine derivatives

**DOI:** 10.3389/fmolb.2023.1288652

**Published:** 2023-11-22

**Authors:** Soukayna Baammi, Achraf El Allali, Rachid Daoud

**Affiliations:** ^1^ Chemical and Biochemical Sciences-Green Processing Engineering, Mohammed VI Polytechnic University, Ben Guerir, Morocco; ^2^ Bioinformatics Laboratory, College of Computing, Mohammed VI Polytechnic University, Ben Guerir, Morocco

**Keywords:** breast cancer, VEGFR-2, 3D-QSAR, ADMET, triazolopyrazine, molecular docking, molecular dynamic simulations, MMPBSA calculation

## Abstract

More people are being diagnosed with resistant breast cancer, increasing the urgency of developing new effective treatments. Several lines of evidence suggest that blocking the kinase activity of VEGFR-2 reduces angiogenesis and slows tumor growth. In this study, we developed novel VEGFR-2 inhibitors based on the triazolopyrazine template by using comparative molecular field analysis (CoMFA) and molecular similarity indices (CoMSIA) models for 3D-QSAR analysis of 23 triazolopyrazine-based compounds against breast cancer cell lines (MCF -7). Both CoMFA (Q^2^ = 0.575; *R* ^2^ = 0.936, R_pred_^2^ = 0.956) and CoMSIA/SE (Q^2^ = 0.575; *R* ^2^ = 0.936, R_pred_^2^ = 0.847) results demonstrate the robustness and stability of the constructed model. Six novel compounds with potent inhibitory activity were carefully designed, and screening of ADMET properties revealed their good oral bioavailability and ability to diffuse through various biological barriers. When compared with the most active molecule in the data set and with Foretinib (breast cancer drug), molecular docking revealed that the six designed compounds had strengthened affinity (−8.9 to −10 kcal/mol) to VEGFR-2. Molecular Dynamics Simulations and MMPBSA calculations were applied to the selected compound T01 with the highest predicted inhibitory activity, confirming its stability in the active pocket of VEGFR-2 over 100 ns. The present results provided the basis for the chemical synthesis of new compounds with improved inhibitory properties against the breast cancer cell line (MCF -7).

## Introduction

The number of worldwide breast cancer diagnoses and deaths was estimated at 2.3 million and 685,000 successively in 2020 with higher incidence rates in older women age groups (WHO, 2020; Muhammad et al., 2022). Despite the clinical utilization of diverse treatment modalities, the current high mortality rates associated with breast cancer persist, particularly in individuals diagnosed with triple-negative breast cancer (TNBC). As a result, there is currently an intensive research initiative aimed at developing innovative and more effective treatments for breast cancer. This goal can be attained through an improved comprehension of the underlying pathophysiology of breast cancer. It is widely acknowledged that angiogenesis represents one of the fundamental characteristics of cancer, particularly in the context of breast cancer. This process is crucial for endothelial cell proliferation, migration, and survival (Chavakis and Dimmeler, 2002). Stimulation of angiogenesis is one of the hallmarks of tumor growth and malignancy (Ziyad and Iruela-Arispe, 2011) and is significantly influenced by vascular endothelial growth factors (VEGFs) and their receptors, vascular endothelial growth factor receptors (VEGFRs) (Roy and Mitra, 2011). VEGF-A, VEGF-B, VEGF-C, VEGF-D, and placental growth factor (PLGF) contribute to the activation of VEGFRs receptors including VEGFR-1 (Flt-1), VEGFR-2 (Flk-1/kinase domain receptor (KDR), and VEGFR-3 (Flt-4) (Shibuya, 2010; Shibuya, 2011).

Several cancers, including colorectal carcinoma, breast carcinoma, and hepatocellular carcinoma, are associated with overexpression of VEGF (Hicklin and Ellis, 2005), which has been linked to the development of oncogenes, lack of tumor suppressor activity, and fluctuations in glucose or oxygen levels (Wedge et al., 2005). To stimulate proliferation of adjacent endothelial cells, VEGF interacts with one of three tyrosine kinase receptors (VEGFR-1–3), and its overexpression is associated with autophosphorylation of VEGFR-2 in malignancy (Wang et al., 2020). Therefore, several lines of evidence suggest that preventing this pathway by inhibiting VEGFR-2 kinase activity impairs angiogenesis and tumor development (Wang et al., 2018).

Several VEGFR-2 modulators have been discovered, some of which are currently in clinical trials (Aziz et al., 2016). However, these inhibitors fail in clinical trials due to acquired resistance and their side effects, such as receptor redundancy, thromboembolic complications, proteinuria, hemorrhage, anal fistulas, gastrointestinal perforations (GI), posterior reversible encephalopathy syndrome, hand-foot skin reaction, oral mucositis, diarrhea, thyroid dysfunction, bone marrow suppression, emergence of hypoxia-tolerant tumor cells, selection of hypoxia-resistant malignant clones, increase in circulating nontumoral proangiogenic substances, and endothelial cell polymorphisms (Modi et al., 2020).

To develop new compounds with potent inhibitory activity, 3D-QSAR and pharmacophore modeling approaches are commonly used to design new ligand-based drugs based on a series of assays and syntheses of different analogs of the compound’s structure (Nicolaou et al., 2008; Acharya et al., 2011). The 3D-QSAR methods such as CoMFA (Comparative Molecular Field Analysis) and CoMSIA (Comparative Molecular Similarity Indices Analysis) have been used to generate valid and consistent models for the synthesis of different compounds and evaluation of their activities on therapeutic targets (Zhao et al., 2011). The major obstacle in drug discovery is the lack of ADME (absorption, distribution, metabolism, and excretion) features (Vugmeyster et al., 2012). To address this problem, a computational approach for drug design that incorporates ADMET predictions is used to generate new hits during the development process (Wu et al., 2020).

In this study, 3D-QSAR methods are used to model and predict the anti-cancer activities of new Triazolopyrazine analogs. Molecular docking analysis and molecular dynamics simulations show that the proposed molecules exhibit greatly enhanced binding and inhibitory activity against VEGFR-2, the major breast cancer receptor, compared to the leading known breast cancer drug, Foretnib. The present results provided the basis for the synthesis of novel triazolopyrazine analogs as improved drugs against breast cancer.

## Materials and methods

### Data collection

A database of twenty-three compounds, including triazole-pyrazine molecules, with their inhibitory activity against breast cancer cell lines (MCF-7), was compiled (Liu et al., 2022). This database was divided into two units for the 3D-QSAR study. Nineteen molecules were randomly selected to be used as training sets for the generation of 3D-QSAR models, and the remaining four molecules were used to evaluate the accuracy of the proposed models. This database was selected for the following reasons: (i) The pIC_50_ values for the compounds ranged from 4.72 to 5.97; (ii) In the field of therapeutic medicine, triazolopyrazine is the active class I pharmacodynamic structure that exhibits good antitumor activity. The IC_50_ values of the twenty-three 1,2,4-triazole compounds used in this study were converted to pIC_50_ using the following expression: (pIC_50_ = −log IC_50_) and presented in Supplementary Table S1.

### Molecular alignment

**FIGURE 1:**
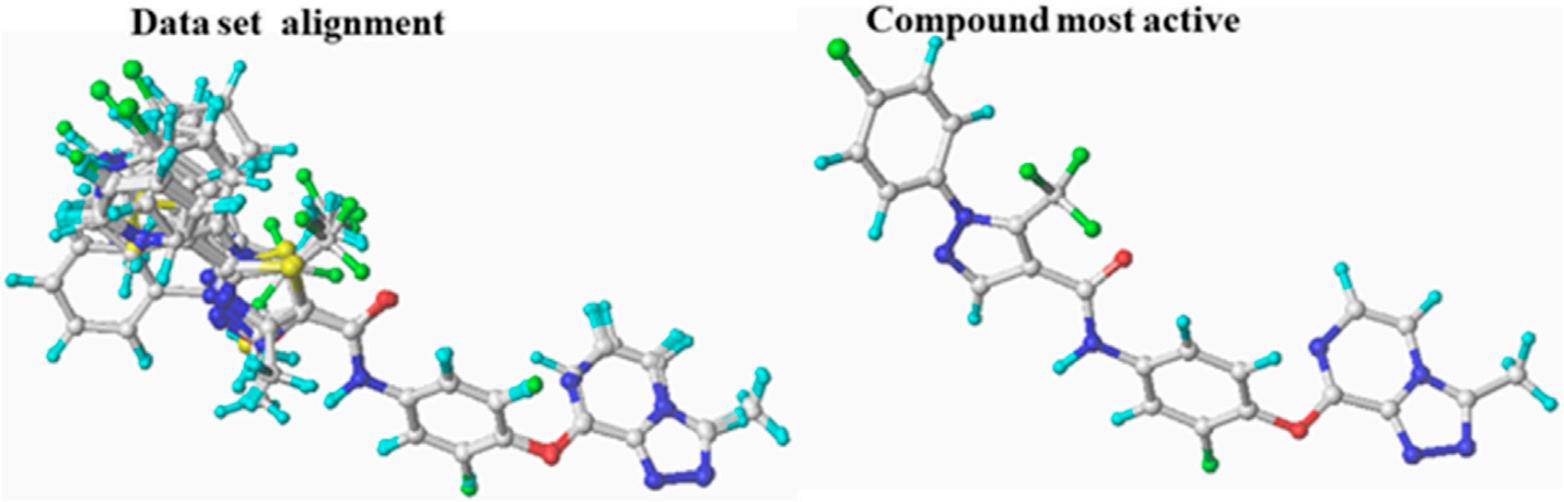
Superposition and alignment of the investigated compounds using molecule 22 as a template.

The molecular modeling studies in this study were performed using the SYBYL-X2.0 package on a Windows 10 64-bit desktop computer (Wang et al., 2018). SKETCH, a module of the SYBYL program, was used to carefully build the 3D scaffolds of the triazolopyrazine derivatives, and the resulting structures were then minimized using the Tripos force field (Waller et al., 1996). The Gasteiger-Hückel atomic partial charges were calculated using the Powell method and a convergence criterion of 0.01 kcal/mol during the minimization phase (Tsai et al., 2010). After structure design and minimization, the distill component of SYBYL-X2.0 performed molecular alignment on the database using molecule 22 (the most active) as a reference (Tabti et al., 2022) (Figure 1).

### 3D-QSAR modeling

CoMFA and CoMSIA techniques were implemented using the Sybyl X-2.0 program (Vistoli and Pedretti, 2007). The CoMFA method was developed using electrostatic and steric fields as well as the Lennard Jones and Coulomb potentials (Tosco and Mackey, 2017). For the steric and electrostatic energies, we used a sp3 hybridized carbon atom with a Van Der Waals radius of 1.52 and a net charge of + 1.0, using the standard rate of 30 kcal/mol for the power cutoff calculations (Hadni and Elhallaoui, 2020). In addition to steric and electrostatic fields, the CoMSIA method was developed to calculate hydrophobic, hydrogen bonding donor, and acceptor fields. The CoMFA model considers the same factors (Roy et al., 2015).

### Partial least square analysis

The 3D QSAR models were generated using the partial least squares (PLS) method, an extension of multiple regression analysis (Vistoli and Pedretti, 2007). For cross-validation, the leave-one-out method (LOO) was first applied (el Mchichi et al., 2022). Here, a single inhibitor was removed from the data set and the derived model was used to make predictions about the activity of that inhibitor. PLS with leave-one-out cross-validation is used to evaluate the accuracy of the model by assessing the optimal number of components (ONC) and the correlation coefficient of cross-validation (Q^2^) (Consonni et al., 2009). The leave-one-out cross-validation method was used to calculate the coefficient of determination (*R* ^2^), the F value (F), and the standard error of the estimate (SEE) (Bouamrane et al., 2022). In addition, to further investigate the robustness of the developed models, they were subjected to external validation with a set of four molecules (Hajjo et al., 2010). The following equation is used to determine the coefficient of determination (R_ext_^2^) of the test set:

$$R_{\text{test}}^{2}=1-\frac{\sum_{i=1}^{test}{\left(Y_{i\;Obs\left(\text{test}\right)}-Y_{i\;Pred\left(\text{test}\right)}\right)}^{2}}{\sum_{i=1}^{training}{\left(Y_{i\;Obs\left(\text{test}\right)}-{\bar{Y}}_{i\;training}\right)}^{2}}$$

Models are considered acceptable if each of the following criteria is met simultaneously: Q^2^ > 0.5, *R* ^2^ > 0.6, R_ext_^2^ > 0.6 (Tropsha et al., 2003).

### Y-randomization test

The Y-randomization technique was applied to the derived models as an additional check to eliminate the effects of randomness (Rücker et al., 2007). After each iteration, the pIC_50_ values of the studied compounds are randomly shuffled several times to obtain a new set of Q^2^ and R^2^. QSAR models are reliable when their Q^2^ and R^2^ values are low, indicating that the excellent calibration result is not due to random correlation (Roy and Mitra, 2011).

### Applicability domain (AD)

Since all QSAR models are developed based on a limited number of molecules, there is a specific region of chemical space where the QSAR model can reliably predict new compounds (Roy et al., 2015). This region is referred to as the application domain (AD) (Cherkasov et al., 2014). Therefore, the accurate application of QSAR models requires the calculation of AD (Sahigara et al., 2012). In this work, we applied a method to define AD based on solving the following equation to determine the effects of different leverage values for all compounds $\left(I=1,2,\ldots ,n\right)$ (Netzeva et al., 2005).

h i=x tiX TX ‐1

X is the descriptor matrix of the training set, and Xi is the descriptor vector of compound i.

The diagram validates the QSAR model if the calculated leverage value (h) is smaller than the critical value of leverage (h*) (Qin et al., 2017).

$$h^{*}=3\left(P+1\right)/n$$

**P** is the number of descriptors and **n** is the number of compounds.

### Molecular docking study

A molecular docking study was performed to predict the molecular interactions between the active site of the VEGFR-2 target protein and the newly designed molecules (Meng et al., 2011). The 3D structure of the VEGFR-2 target protein (PDB code: 4ASD) was obtained from the Protein Data Bank (www.rcsb.org) (Burley et al., 2019). Autodock tools were used to prepare the protein before docking (Hernández-Santoyo et al., 2013). Water molecules and co-crystallized small molecules were removed from the protein structure, the polar hydrogen and Kollmann charges were added to the structure (Shivanika et al., 2020). The grid box spacing was set to 0.375 Å, the center to (−24.611 Å, −0.388 Å, −10.929 Å), and the lattice size to 20 Å × 20 Å × 20 Å where the co-crystallized ligand interacts with the active residues (Baammi et al., 2023a). Nine poses were constructed for each protein-ligand complex based on docking affinity. The Discovery Studio Viewer was used to display and analyze the docking results to find the important interactions between the ligands and the protein binding site (Adeniji et al., 2020; Kesari et al., 2020; Singh et al., 2022).

### Redocking

Docking accuracy was determined by comparing the root-mean-square deviation (RMSD) of the heavy atoms between the docked pose and the crystallographic pose of the ligand (sorafenib) (Wen et al., 2019). If the RMSD is below 2 Å, a molecular docking approach is robust (Baammi et al., 2023b).

### Molecular dynamics simulation

To determine the structure-function relationship, MD simulations were performed using the GROMACS 2019.3 software program (Abraham et al., 2015) on the docked complexes of compound T01, the active molecule (molecule22), and Foretinib. The CHARMM27 force field was used for the protein (Lindahl et al., 2010), and the topology for the ligands was generated using the Swissparam server (Zoete et al., 2011). Prior to neutralization in the system with counterions, each complex was resolved in a dodecahedron box (1.0 nm) using the TIP3P water model, and counterions (Na^+^) were added to neutralize the system (Saini et al., 2021). The steepest descent method was used to achieve both the minimum energy and maximum force, with Fmax set at 1000 kJ/mol/nm (Baammi et al., 2022). To equilibrate the system at 300 K and 1 bar, two 100 ps simulations were performed in rapid succession using canonical NVT and isobaric NPT ensembles. Subsequently, 100 ns molecular dynamics simulations were performed for each molecule. The output trajectories were generated, and the data files were analyzed to better understand the behavior of the protein.

## Results and discussion

### Results of CoMFA and CoMSIA

The main objective of this step is to build powerful CoMFA and CoMSIA models based on the observed and estimated pIC_50_ values of different models used for training and test sets (Supplementary Table S2) (Sharma et al., 2016). To create the CoMFA model, steric and electrostatic fields were combined. Meanwhile, thirty one different combinations of the five fields: steric, electrostatic, hydrophobic, H-bond donor, and H-bond acceptor were applied to build the CoMSIA models (Supplementary Table S3) (Lu et al., 2011). Table 1 displays the results produced using the CoMFA and CoMSIA models. The Q^2^ from the cross-validation provides information about the robustness of the CoMFA and CoMSIA models. If Q^2^ ≥ 0.3, the built model is only significant at the 5% level, while Q^2^ ≥ 0.5 means that the model is statistically significant (Chu et al., 2020).

**TABLE 1:** Summary of 3D-QSAR results.

Models	R^2^	Q^2^	SEE	F	NOC	R_pred_^2^	Fractions				
							Ster	Elec	Hyd	Don	Acc
CoMFA	0.936	0.575	0.102	29.265	6	0.956	0.64	0.36	-	-	-
CoMSIA	0.938	0.575	0.100	30.437	6	0.845	0.642	0.358	-	-	-

**FIGURE 2:**
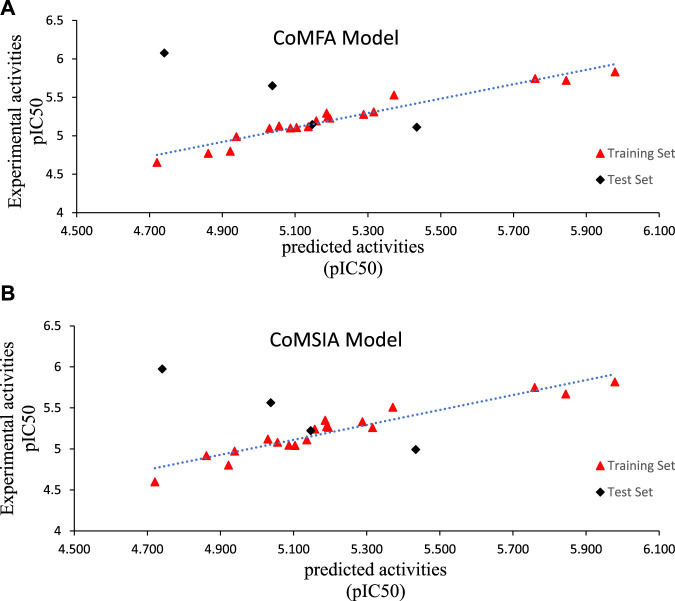
Observed versus predicted pIC_50_ of training and test sets of Triazoloyrazine derivatives inhibitors based on **(A)** CoMFA and **(B)** CoMSIA models.

For the CoMFA model, PLS regression analysis yielded a high *R* ^2^ (0.936), F values of 29.265, a cross-tested coefficient of determination Q^2^ (0.575), a standard error estimate (SEE) of 0.102, and a small optimal number of components N (6). The CoMFA model was validated using four triazolopyrazine compounds yielding an R_pred_^2^ of 0.956. The steric and electrostatic fields contributed 64% and 36%, respectively, to the CoMFA model, with the electrostatic field being more influential compared to the steric field. For the CoMSIA model, the values of Q^2^, R^2^, F, SEE, and N were 0.575, 0.938, 30.437, 0.100, and 6, respectively, and the resulting model exhibited a high level of internal predictability. The same parameters were used to validate the CoMSIA/SE and CoMFA models. The percentage contributions of the steric and electrostatic fields were 64.2% and 35.8%, respectively. The consistency of the CoMSIA models was demonstrated by an external validation correlation coefficient (R_pred_^2^) of 0.845 > 0.6, indicating a high level of accuracy (Chirico and Gramatica, 2011). Furthermore, all these results showed that the steric field is the most important factor in the development of new anticancer drugs. The correlation between observed and predicted activity for the CoMFA and CoMSIA models (Figure 2) exhibits a satisfactory linear correlation for all molecules except molecule 22.

### Applicability domain (AD) and Y- randomization test

**FIGURE 3:**
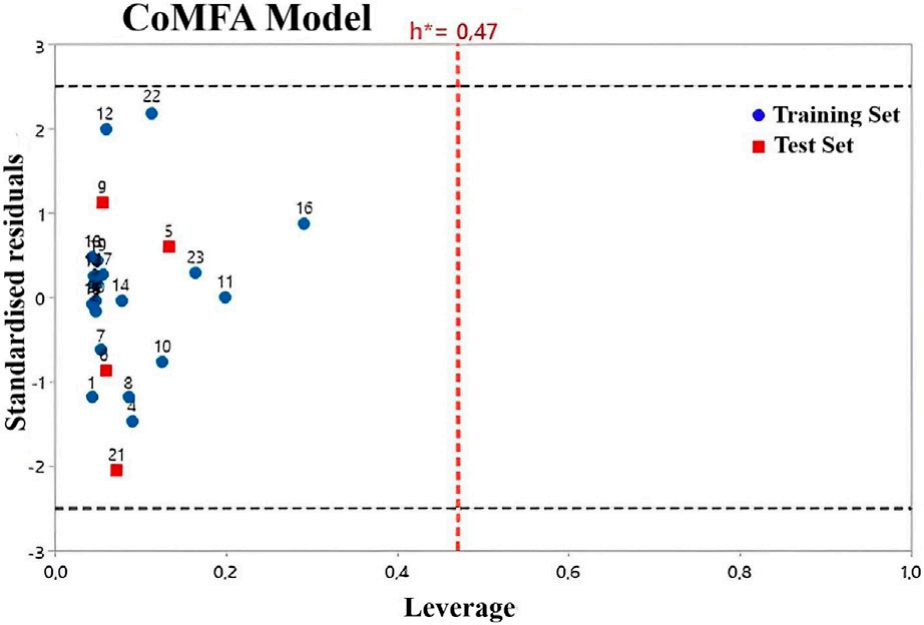
Williams plot for CoMFA/ES model.

**FIGURE 4:**
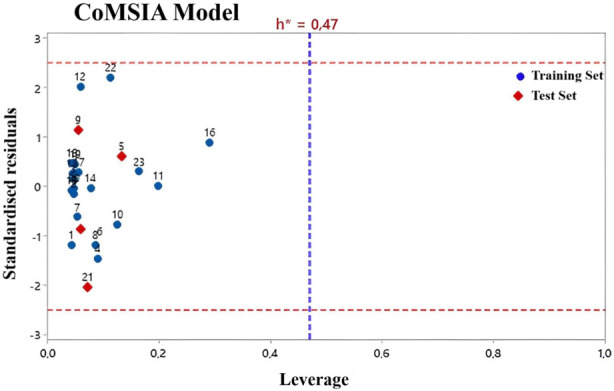
Williams plot for CoMSIA/ES model.

William’s plot refers to a graph that compares the leverage values and standardized residuals of a particular group of compounds (Beheshti et al., 2016). There are other methods to define the applicability domain models because, without defining the scope, it is not possible to use any of the QSAR models to predict the activities that the new compounds would perform (Roy et al., 2015). Compounds with leverage greater than the threshold are considered potentially disruptive to the performance of the model and are therefore flagged as influential (Moussaoui et al., 2023; Soufi et al., 2023). In this study, the AD of the CoMFA and CoMSIA QSAR models is defined using William’s plots (Figures 3, 4). For the developed CoMFA/ES and CoMSIA/SE models, the warning leverage (h*) was calculated as 0.47. Our result shows that neither the training nor the test sets contain any compounds that are particularly outliers, and all compounds have leverage values lower than the warning h* value (hi < h*). Hence, the model has such good predictive capabilities and robust statistical parameters (Benfenati et al., 2007). In addition, the Y-randomization test was used to examine the robustness of each model. After each iteration, the independent variables of the studied compounds (pIC_50_) are randomly shuffled (Edraki et al., 2016). The results of 20 random shuffles for the Y-randomization test are shown in Supplementary Table S4 and Supplementary Table S5. After this test, both models have lower values for Q^2^ and R^2^ compared to our original models, indicating that the developed models are very robust and reliable and not due to chance.

### Contour maps analysis of CoMFA and CoMSIA

**FIGURE 5:**
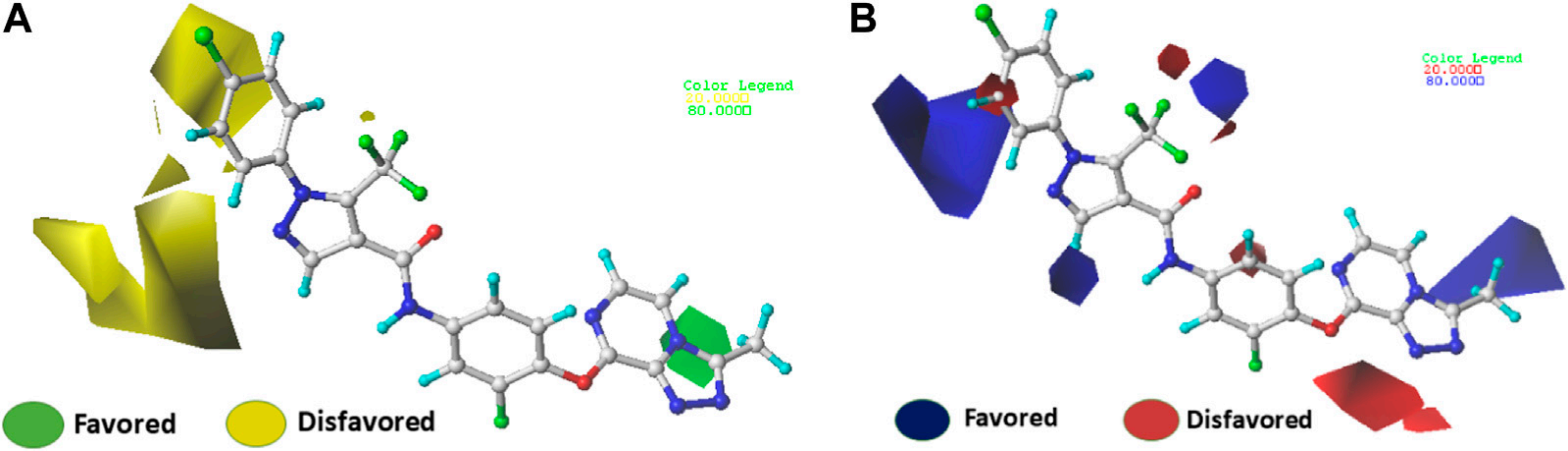
Electrostatic **(A)** and steric **(B)** contour maps of the CoMFA model around molecule 22. When the peripheral part of the molecule is colored green, the activity of the molecule is enhanced by a group with a large connecting space, and when it is colored yellow, the activity of the compound could be reduced by the presence of the group with the large connecting space. The blue colored regions indicate a preference for positive groups that may lead to an increase in anti-VEGFR-2 activity, while the red colored regions indicate a preference for negative groups.

**FIGURE 6:**
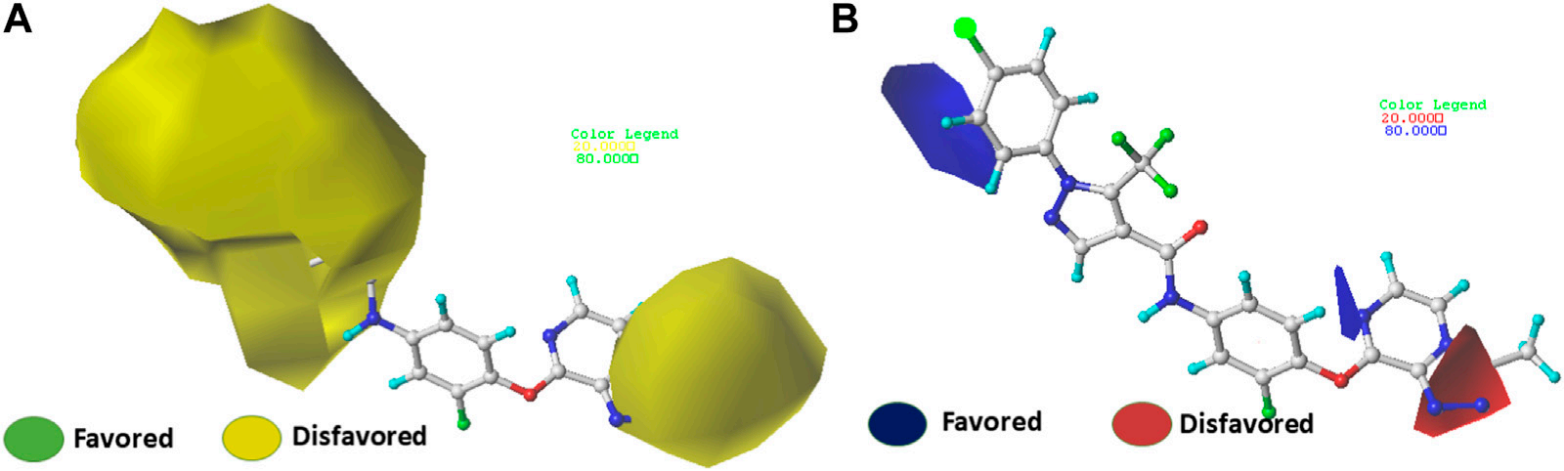
Electrostatic **(A)** and steric **(B)** contour maps of the CoMSIA model around molecule 22. When the peripheral part of the molecule is colored green, the activity of the molecule is enhanced by a group with a large connecting space, and when it is colored yellow, the activity of the compound could be reduced by the presence of the group with the large connecting space. The blue colored regions indicate a preference for positive groups that may lead to an increase in anti-VEGFR-2 activity, while the red colored regions indicate a preference for negative groups.

The use of 3D contour maps to represent the QSAR equation is an effective way to illustrate the relationships between VEGFR-2 inhibitors and their anti-cancer activities. The 3D contour maps were made using the Stdev* Coeff field type. Due to its high activity, molecule 22 (Figures 5, 6) was selected as a representative compound to analyze the performance of CoMFA and CoMSIA models. Figure 5 shows a contour map of the steric field of CoMFA, with the effect of the steric field on the activity depicted in green and yellow. This steric field accounts for 64% of the total contribution.

Figures 5A shows a green contour dispersed around the R1 substituent, suggesting that adding more groups at the R1 substituent site could increase the activity of the compound. For example, the bioactivity of compound 12 (pIC_50_ = 5.75) with a methyl ring was significantly higher than that of compound 1 with a hydrogen ring (pIC_50_ = 4.86). Figures 5B displays the contour map of the electrostatic field of CoMFA. The blue (80%) and red (20%) colors represent the influence of the electrostatic field (36%) on the activity. The compound of an electron-donating group enhances the activity of the compound, as shown by the blue contour around the molecule, while the compound of an electron with drawing group also decreases the activity of the compound, as shown by the red contour. The activity of the molecule increases when electron-withdrawing groups are placed near the X substituent (Supplementary Table S1), which explains the higher activity of compound 9 compared to compound 3 (pIC_50_ = 5.43 vs. pIC_50_ = 5.19). The same observation was made when the hydrogen was replaced by a fluorine atom (compounds 16 pIC_50_ = 4.86 vs. compound 6 pIC_50_ = 5.03)

The contour maps of the steric field (Figures 6A) or the electrostatic field (Figures 6B) of CoMSIA are nearly identical to those of the CoMFA model. Thus, the CoMSIA fractions (Table 1) indicate that the major fields potentially characterizing inhibitory activity are steric (64.2%) and electrostatic (35.8%), which are the same fields found in COMFA.

### Newly designed compounds

**FIGURE 7:**
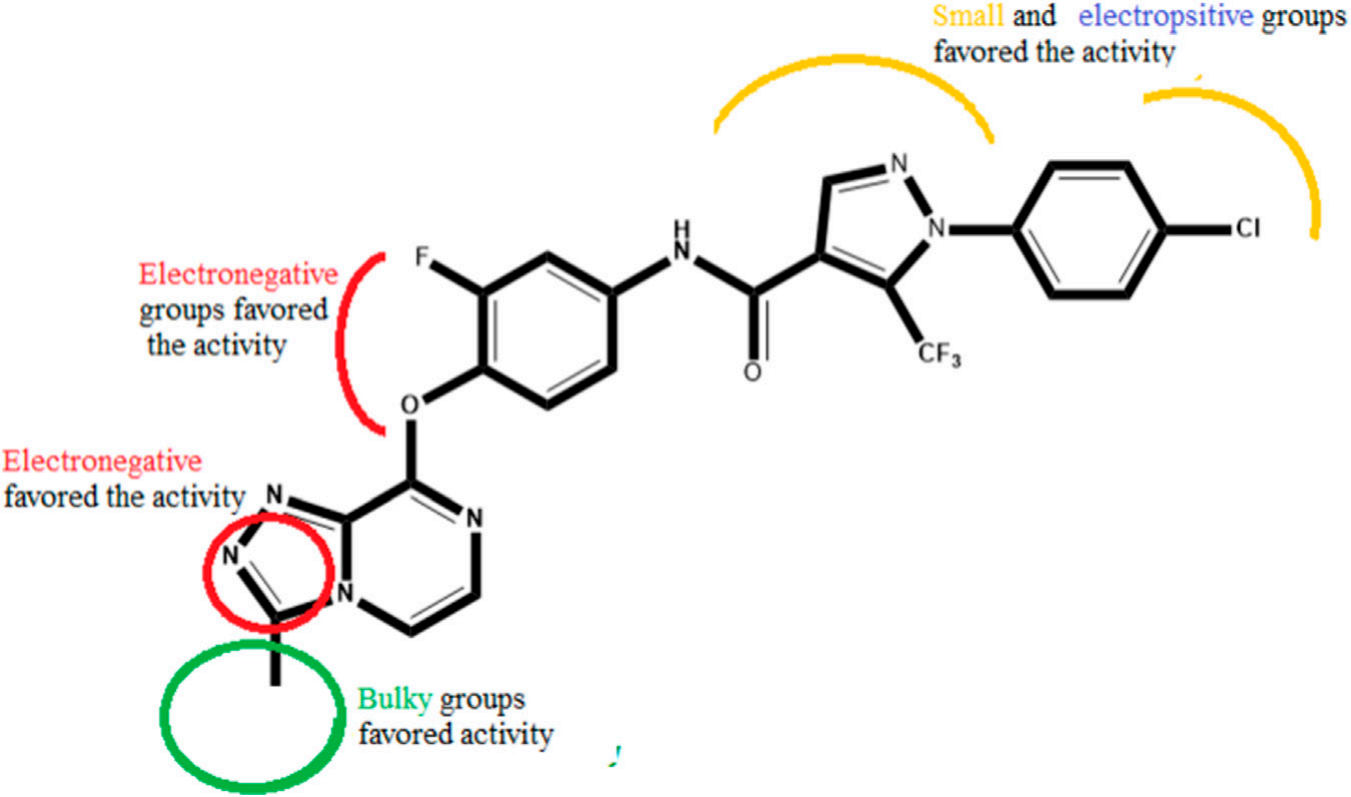
The 3D-QSAR analysis revealed new insights into the structure-activity relationship.

Several novel triazolopyrazine derivatives were designed to increase activity considering the 3D QSAR results and the predominant modified region determined by the contour map results, using molecule 22 as a template (Figure 7).

In the case of the newly predicted molecules T01, T02, and T03, the replacement of methyl by isopropyl, phenyl, and cyclobutyl, respectively, in R1 increases the activity (Table 2). In addition, simultaneous substitution of R1 and radical Z increases the activity from 5.83 to 6.35, which is the case for T04 and T06; A4 (R1 = isobutyl + Z = fluorite); T05 (Z = pyrroline fluoride); and T06 (R1 = isobutyl + radical Z = fluorine). All these molecules blocked VEGFR-2 much better than compound 22, suggesting that they could be used as new VEGFR-2 inhibitors.

**TABLE 2:** The 3D-QSAR model’s predicted activity pIC50 (Pred).

Compounds	Structures	Activity estimated pIC_50_		Binding affinity (Kcal/mol)
		CoMFA	CoMSIA	
T01	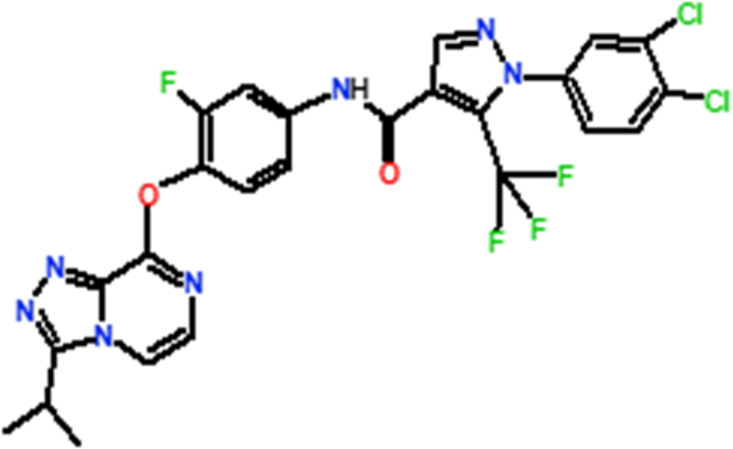	6.430	6.594	−9.6
T02	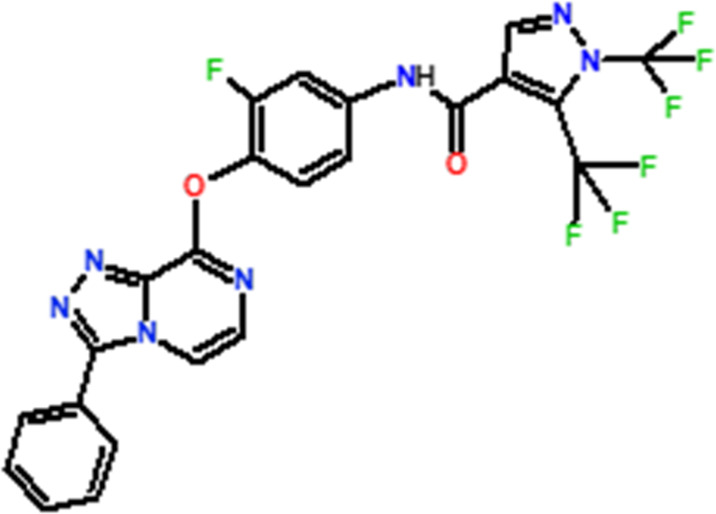	6.182	6.263	−9.7
T03	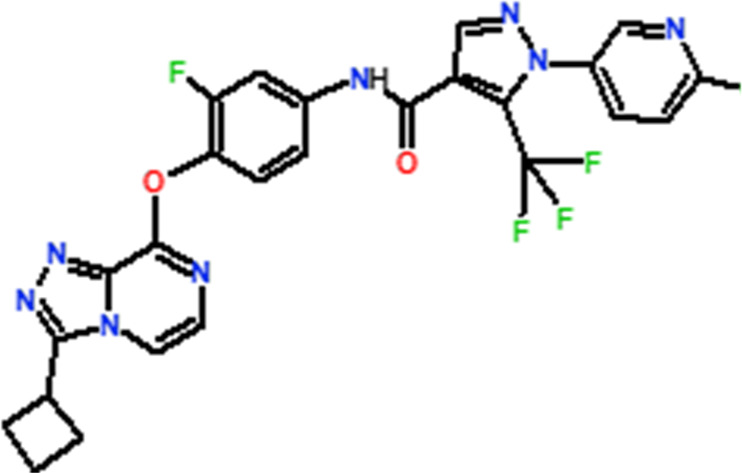	6.166	6.502	−10
T04	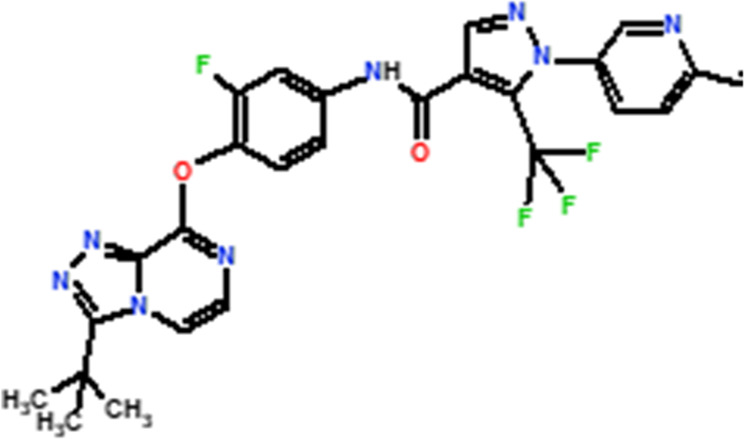	6.357	6.614	−8.9
T05	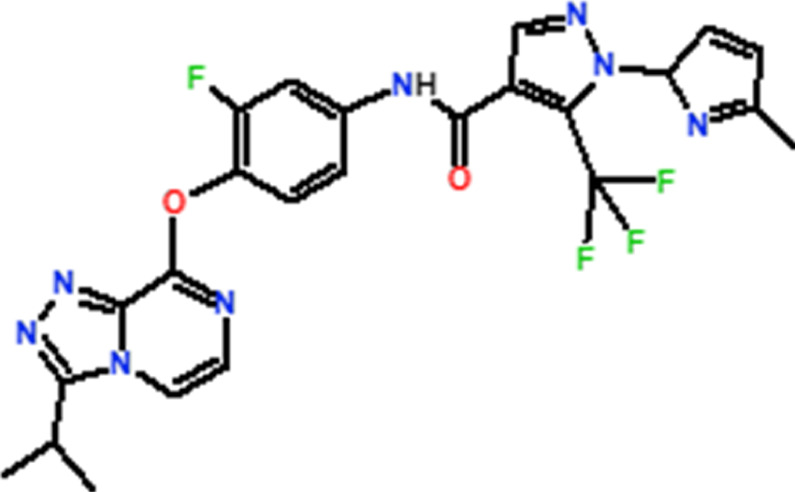	5.808	6.482	−9.8
T06	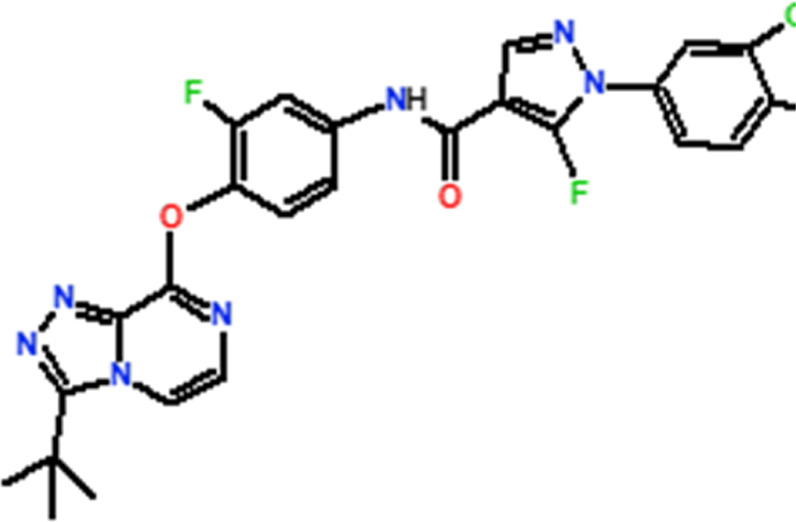	6.136	6.368	−10
22	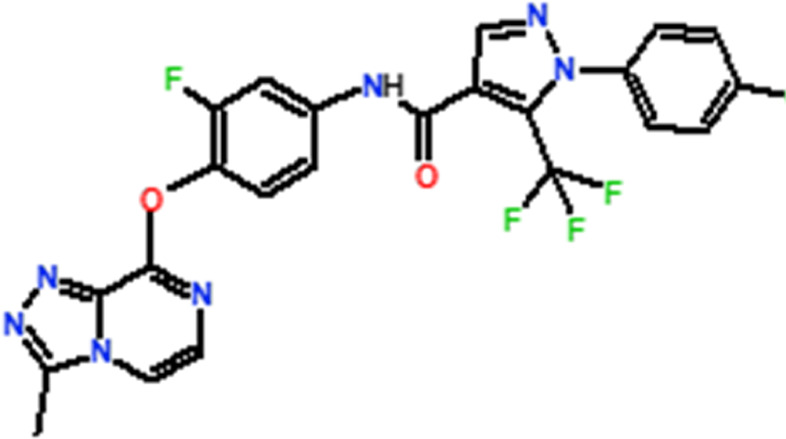	5.83	5.82	−8.8

### ADME/toxicity prediction and analysis

Due to the lack of permeability of the blood-brain barrier, toxicity problems, and lack of efficacy, most drugs fail in the research and development phase. Therefore, prediction and optimization of ADMET properties of novel chemical entities are crucial to avoid potential problems during clinical trials. pKCSM (Pires et al., 2015) and the Swiss ADME (Daina et al., 2017) Predictor are commonly used programs to predict ADMET properties of small molecules. The *in silico* ADMET results for the six newly designed compounds are given in Table 3.

**TABLE 3:** ADME parameters of newly designed compounds.

Compounds	Absorption			Distribution		Metabolism							Excretion
	Water solubility (Log mol/L)	Caco2 permeability (%) Absorbed	Intestinal absorption (human)	BBB permeability (Log BB)	CNS permeability (Log PS)	Substrate		inhibitor					Total Clearance (Log ml/min/kg)
						CYP							
						2D6	3A4	1A2	2C19	2C9	2D6	3A4	
T01	−3.393	0.374	98.24	−2.05	−2.824	No	Yes	No	No	Yes	No	Yes	0.267
T02	−3.364	0.129	96.121	−2.167	−3.218	No	Yes	No	No	Yes	No	Yes	−0.617
T03	−3.12	1.278	100	−2.157	−3.4	No	Yes	No	No	No	No	Yes	−0.458
T04	−3.839	0.735	87.94	−1.858	−3.127	No	Yes	No	Yes	No	No	Yes	−0.383
T05	−3.662	0.482	97.039	−1.998	−3.033	No	Yes	No	Yes	Yes	No	Yes	−0.466
T06	−3.425	0.479	90.46	−1.834	−3.011	No	Yes	No	Yes	Yes	No	Yes	−0.495
Molecule 22	−3.265	0.797	94.765	−1.915	−3.154	No	Yes	No	No	No	No	Yes	−0.01

The *in silico* ADMET results for the six newly designed compounds are given in Table 3. The ability of a drug to be absorbed into the bloodstream after being administered is directly related to its water solubility. Absorption values for all compounds are greater than 70% (100% for T03), indicating a greater absorption potential in the human intestine (Table 3). A drug travels to various organs and systems once it enters the bloodstream. Because of the blood-brain barrier, most substances are unable to cross over from the bloodstream into the central nervous system. This barrier is the primary one that stands between the bloodstream and the CNS. In order to enter the central nervous system (CNS), a drug molecule must therefore satisfy a number of requirements before it can cross the blood-brain barrier (BBB). These prerequisites include blood-brain permeability surface product (logPS) greater than −2 and a logarithmic ratio brain-to-plasma drug concentration (logBB) greater than 0.3. As shown in Table 1, designed compounds meet these values. In addition, the activity of an isoenzyme may be decreased or increased, depending on the drug. In some cases, the metabolism of a drug requires more than one isoenzyme. Approximately 90% of commonly prescribed drugs require the involvement of four isoenzymes for their metabolism. These isoenzymes are referred to as CYP1A2, CYP2C9, CYP2D6, and CYP3A4.

The in-silico Swiss-ADME prediction shows that all expected compounds are both substrates and inhibitors for 3A4, but not for 2D6. The results of this assay showed that none of the chemicals were mutagenic or carcinogenic. In addition, the acute toxicity (LD50) of the new compounds ranged from 2.423 to 2.923 mol/kg, and none of the candidates caused skin sensitization (Table 4). These proposed compounds are reliable candidates for further clinical studies because they exhibit exciting properties such as high intestinal absorption, distribution, permeability, and toxicity across the blood-brain barrier.

**TABLE 4:** Toxicity profile of newly designed compounds.

Compounds	AMES toxicity	Max. tolerated dose (human)	hERG I/II inhibitor	Oral rat acute toxicity (LD50)	Oral rat chronic toxicity (LOAEL)	Skin sensitization
	Yes/No	Log mg/kg/day	Y/N	Mol/kg	Log mg/kg_ bw/day	Yes/No
T01	No	0.552	No/Yes	2.648	−1.075	No
T02	No	0.547	No/Yes	2.923	−0.985	No
T03	No	0.534	No/Yes	2.625	−0.454	No
T04	No	0.423	No/Yes	2.423	−0.041	No
T05	No	0.285	No/Yes	2.488	−0.426	No
T06	No	0.482	No/Yes	2.649	−0.843	No
Molecule 22	No	0.603	No/Yes	2.729	−0.426	No

### Docking results

The binding pattern of the novel designed inhibitors to VEGFR-2 was analyzed using molecular docking and compared to the mode of action of the standard inhibitor (Foretinib). To validate our approach and parameters before docking all chemicals, we redocked the native ligand sorafenib of VEGFR-2 into the binding pocket. The redocked conformation of sorafenib is very similar to its original conformation; the RMSD between the two configurations is only 1.2 to 1.01 (Supplementary Figure S1). Once the binding site was determined, docking was performed for each compound, including the active compound, proposed compounds, and Foretinib using the identical docking settings. The 3D and 2D binding interactions of all compounds are presented in Table 5.

**TABLE 5:** The 3D and 2D binding interactions showed that all proposed molecules interacted with a higher number of residues via hydrophobic interactions than Foretinib and compound 22.

Complex	3D binding interactions	2D binding interactions
*Foretinib-VEGFR-2*	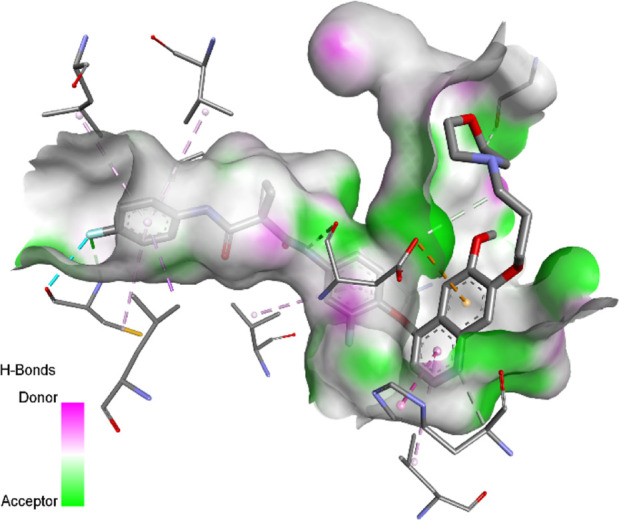	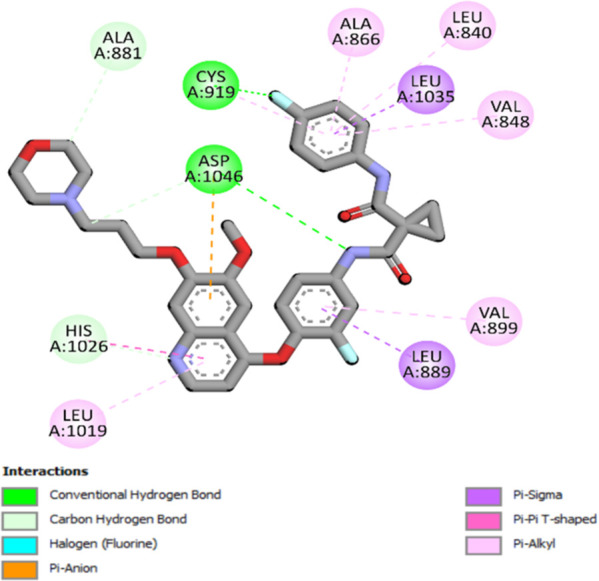
*MOL22-VEGFR-2*	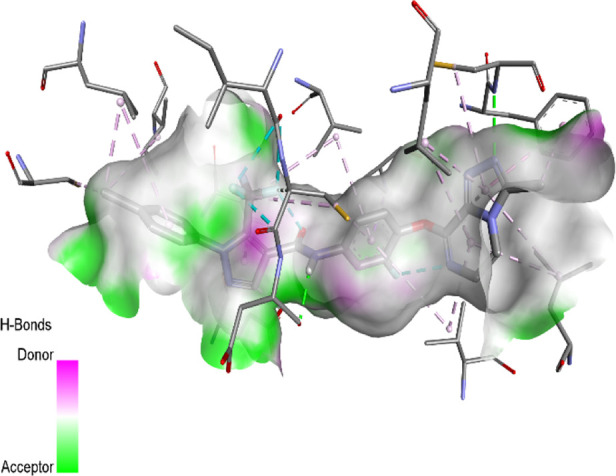	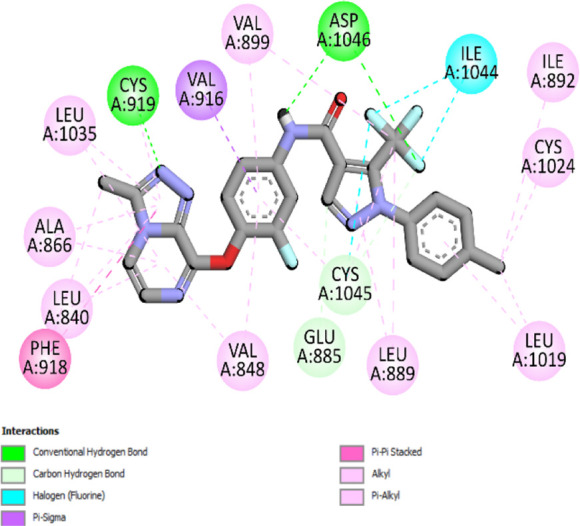
T01-VEGFR-2	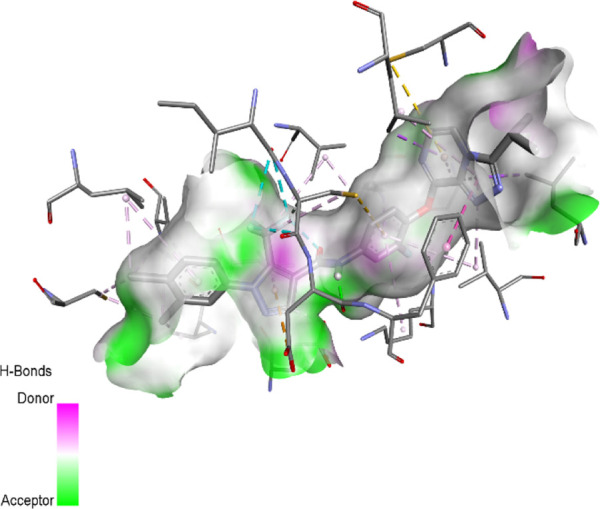	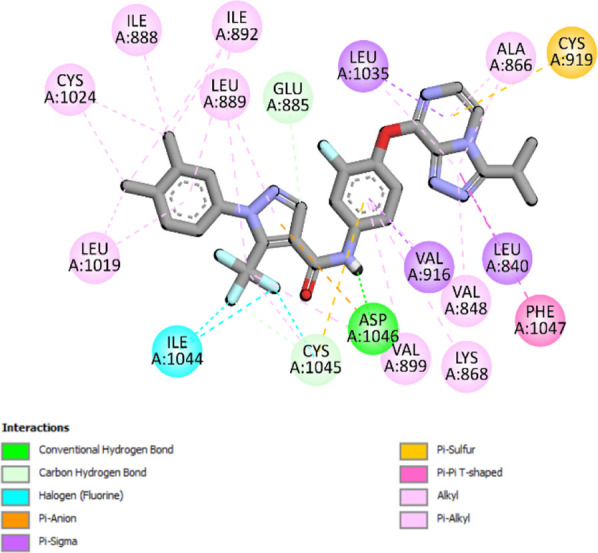
*T02-VEGFR-2*	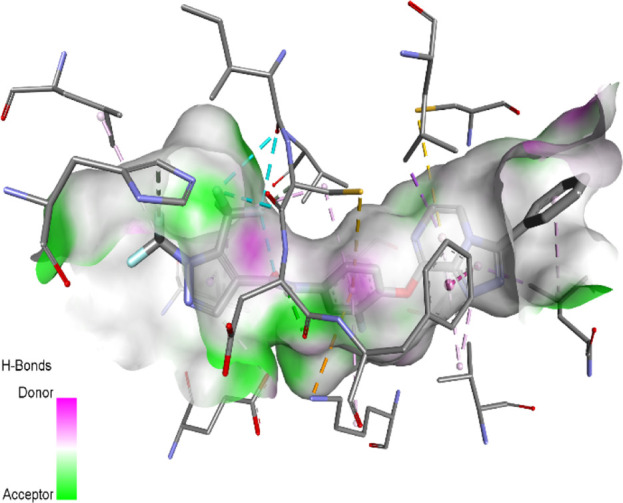	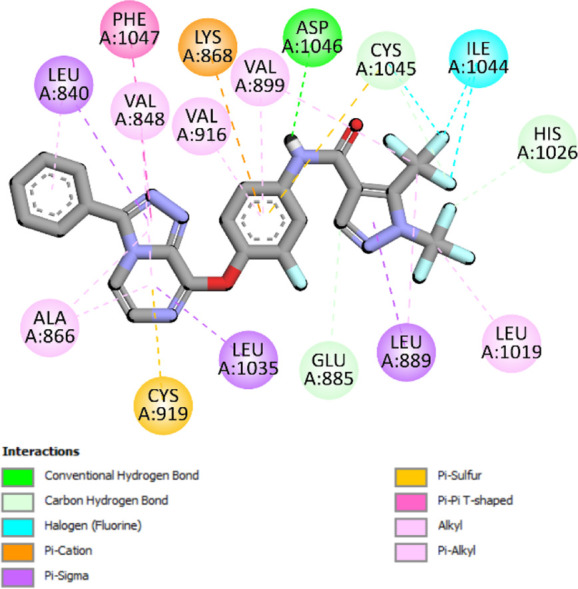
*T03 –VEGFR-2*	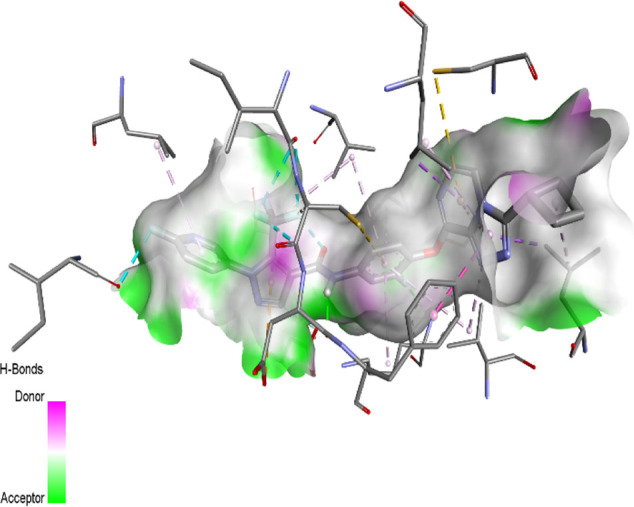	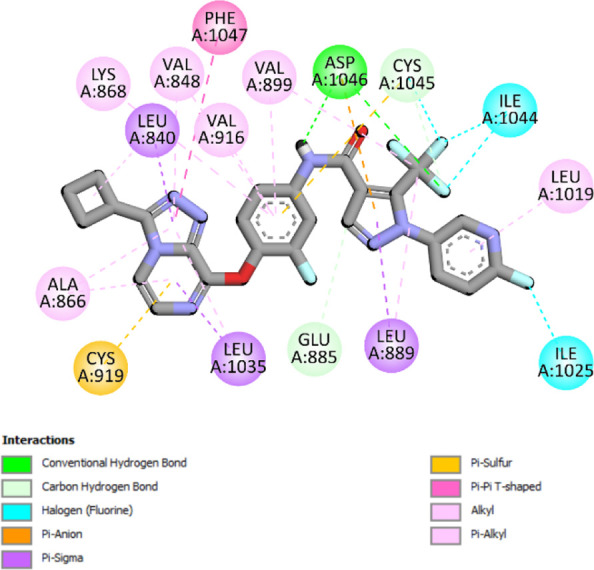
*T04- VEGFR-2*	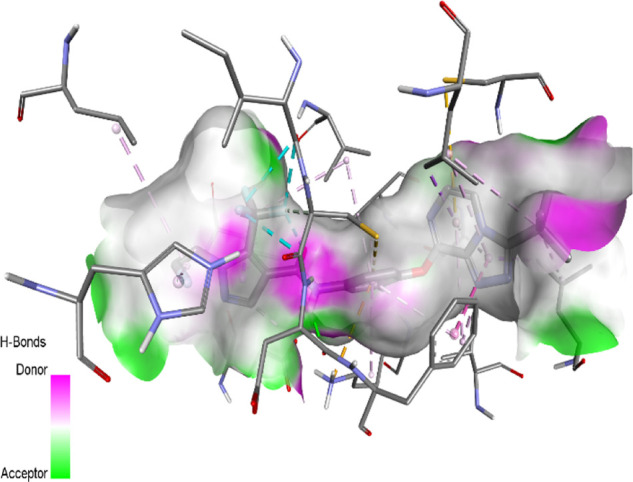	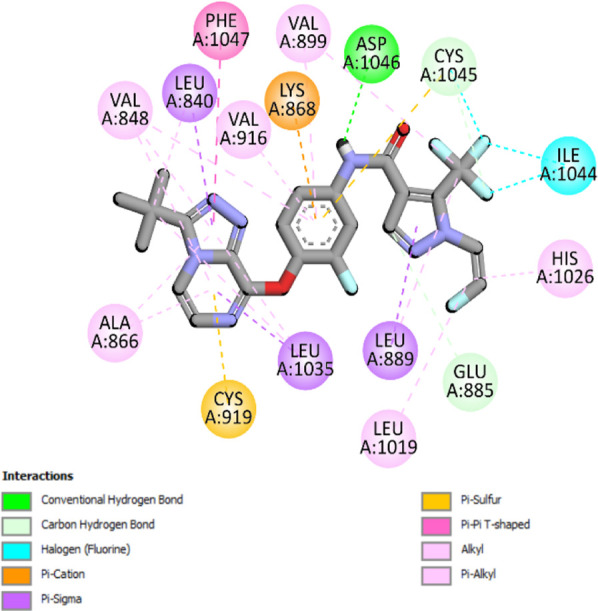
*T05- VEGFR-2*	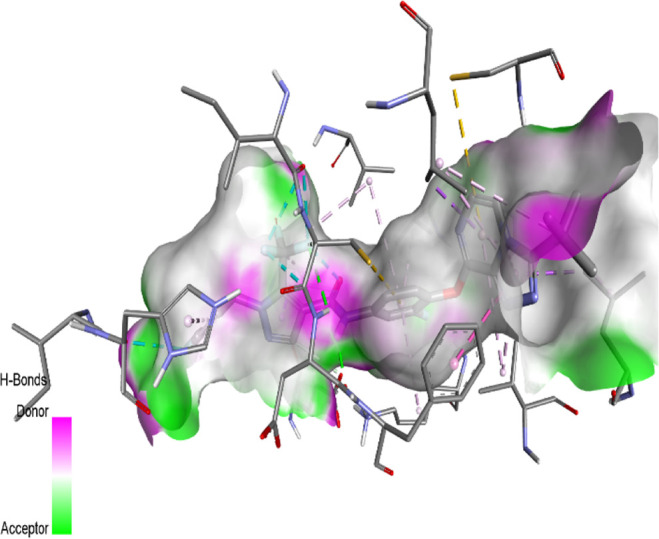	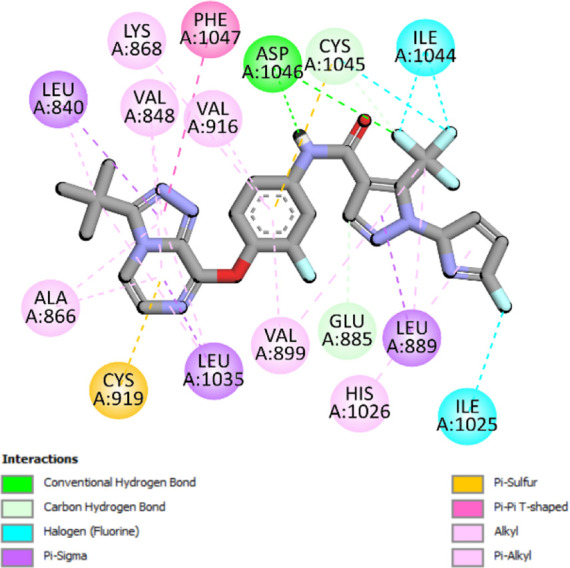
*T06- VEGFR-2*	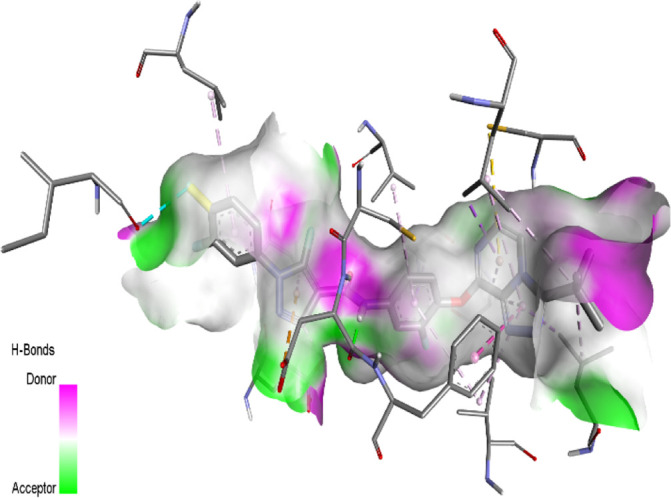	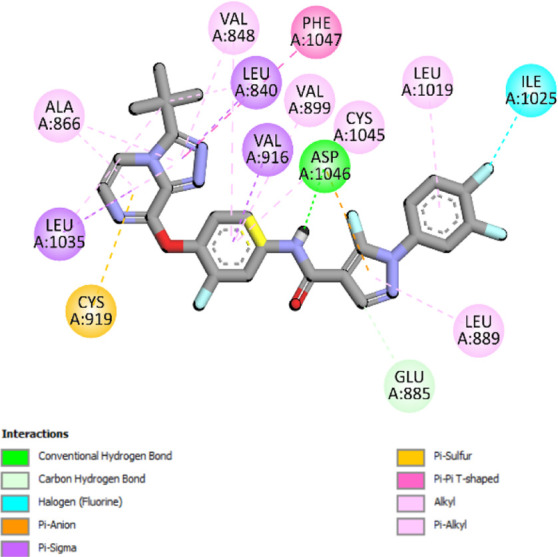

Foretinib, a receptor tyrosine kinase inhibitor, has the potential for treating breast cancer by acting on multiple kinases involved in cancer growth. Compared to Tamoxifen and Trastuzumab, which target specific pathways, Foretinib offers a broader spectrum of activity. It formed two H-bonds during docking with residues Cys919, and Asp1046 (Table 5), and several hydrophobic and electrostatic interactions with Ala866, Leu840, Val848, Val899, Leu889, Leu1019, His1026, Asp1046 and Leu1035. In addition, Foretinib formed three carbon-hydrogen bonds with Ala881, His1026, and Asp1046. Molecule 22, the most active molecule in the data set, showed two conventional H-bond with Asp 1046, and Cys919, two carbon-H bonds due to the strong interaction of Cys1045 and Glu885, twelve hydrophobic via Pi-sigma, Pi-Pi T-shaped, P-alkyl and alkyl interactions with Cyc919, Leu1035, Leu840, Ala866, Phe918, Val848, Leu889, Leu1019, Cy1024, ILe892, Val899, Val916 and two Fluorine interactions with Cys1045 and Ile1044. By the same token, all complexes formed by designed compounds (T01-T06) and the VEGFR-2 receptor have better binding affinity ranging from −9.6 to −10 kcal/mol (Table 2). Therefore, these complexes are more stable than the complexes generated by Foretinib (−9 kcal/mol) and compound 22 (−8.8 kcal/mol) in the data set, suggesting that these compounds have a higher inhibitory potential against VEGFR-2. Furthermore, the three-dimensional binding interaction of the compounds (T1-T6) showed a similar H-binding interaction profile with Asp1046 and hydrophobic and electrostatic interactions with Val899, Val848, Ala866, Leu1035, Leu889, Leu1019, Leu1035, and Leu840, with compound 22, and Foretinib suggesting that these amino acids play a critical role in enhancing activity as reported in previous studies (Yousef et al., 2022; Parves et al., 2023). These compounds interact with a higher number of residues via hydrophobic interactions than molecule 22 and Foretinib, which increases their stability and affinity in the binding pocket of VEGFR-2.

### Molecular dynamics simulation

**FIGURE 8:**
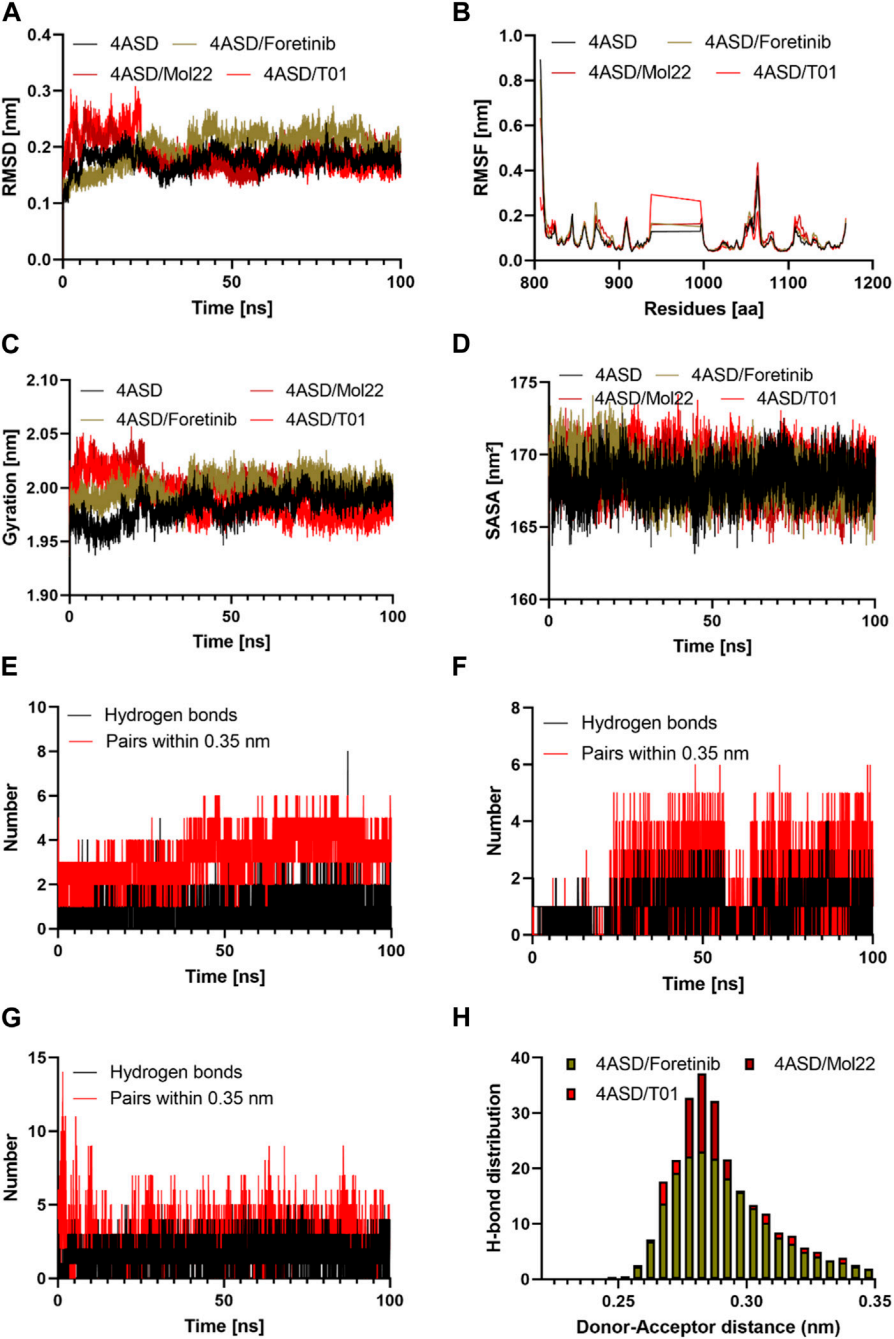
The results of the molecular dynamics study: **(A)** Time evolution of the backbone of the target protein; **(B)** The comparative RMSF values for the target protein with the reference molecule, molecule 22, and designed molecule T01; **(C)** The comparative Radius of gyration values for the target protein with the reference molecule, molecule 22, and designed molecule T01; **(E, F, G)** The comparative hydrogen bonds and pairs within 0.35 nm for the target protein with the reference molecule, designed molecule 20, and designed molecule T01; **(H)** Hydrogen bond distributions of the reference molecule, molecule 22, and the designed molecule T01 with the target protein during the 100 ns. **(D)**: The comparative SASA values for the target protein with reference molecule 22, and designed molecule T01.

Molecular dynamics modeling was used to assess the effects of molecule 22, the lead compound (Foretinib), and designed compound T01 with the highest predicted inhibitory activity (Table 2) on the structure of VEGFR-2 and its stability in the binding pocket (Agrahari et al., 2018; Agrahari et al., 2019). Accordingly, Gromacs 2019.3 was used to conduct a molecular dynamics simulation for these complexes (Figure 8), and various features such as root mean square deviation (RMSD), Root-mean square fluctuation (RMSF), and Radius of gyration (Rg) were examined on the trajectories corresponding data files.

### Conformation of protein

The RMSD was computed during the simulation to establish the overall stability of the selected systems. The calculated value was considered as the main criterion for measuring the convergence of the system. The RMSD values of the 4ASD, 4ASD-Foretinib, mol22, and T01 complexes were calculated to be 0.17, 0.19, 0.18, and 0.18 nm, respectively. Up to 100 ns in the simulation, none of the three systems had a significant shift in root-mean-square-deviation (RMSD) values, which quantify conformational changes over time (Figures 8A). The resulting RMSD plot for 4ASD-Mol22 and 4ASD-T01 showed an increasing trend with increasing RMSD values between 0 and 25 ns, ranging from 0.10 to 0.30 nm, indicating that the compounds were adapting to a new conformation within the binding pocket (Aljuaid et al., 2022). Thereafter, the plateau continued until it reached a final value of 0.20 nm, which is below the threshold value of 0.3 nm. However, 4ASD-Foretinib has the same profile but not the same RMSD values as apo 4ASD. Finally, the lower RMSD values for all complexes studied indicate that the T01 inhibitor in 4ASD is stable, which provides a good basis for our investigation.

### Root mean square fluctuation (RMSF)

Using the RMSF approach, we analyzed how ligand binding alters flexible protein structure and essential amino acid behavior. During simulation, a higher RMSF value indicates greater flexibility, while a lower RMSF value indicates greater rigidity (En-nahli et al., 2022). The RMSFs of the Apo 4ASD, 4ASD-Foretinib, 4ASD-mol22, and 4ASD-T01 complexes were calculated (Figures 8B). Compared with the apo form of 4ASD, the fluctuations of the residues in the ligand-bound complexes are quite stable, especially in the region where the residues participate in ligand binding. Furthermore, the average RMSF values of the Apo 4ASD, 4ASD-Foretinib, 4ASD-mol22, and 4ASD-T01 complexes were 0.10, 0.10, 0.09, and 0.08 nm, respectively. This result indicates that the binding of compound T01 contributes to the structural stability of 4ASD.

### Radius of gyration analysis

We calculated the radius of gyration (Rg) as a function of time to investigate how the compactness of the protein structure changes when bound to various ligands. When Rg is sufficiently high, a ligand tends to be flexible, making it unstable. Conversely, conformations with lower Rg values tend to be dense and tightly packed (Naz et al., 2023). The average Rg values of the Apo 4ASD, 4ASD-Foretinib, 4ASD-mol22, and 4ASD-T01 complexes were 1.98, 2.00, 1.98, and 2.00 nm, respectively, suggesting that the binding of Foretinib, mol22, and the designed compound T01 to the 4ASD packing does not cause a significant change. As can be seen in Figure 8C, the Rg of the 4ASD/T01 complex appears to stabilize more rapidly during the 100-ns simulation than that of the 4ASD-Foretinib and 4ASD-mol22 complexes.

### Solvent accessible surface area

SASA measures the surface area of the protein that is in direct contact with the solvent. The interpretation is based on the fact that the surface of the macromolecule-ligand complex is in contact with the water molecules surrounding it (Baammi et al., 2023b). The change of SASA for the complex of protein active molecule and for the complex of protein-designed molecules was analyzed during 100 ns (Figures 8D). The average values of SASA for the Apo 4ASD, 4ASD-Foretinib, 4ASD-mol22, and 4ASD-T01 complexes were 168.18 nm^2^, 169.00 nm^2^, 168.48 nm^2^, and 168.57 nm^2^, respectively. Analysis of these data sets revealed no significant variation in SASA values between complexes.

### Hydrogen bonds analysis

Hydrogen bonding is an essential feature that determines binding affinity and contributes to the binding relationship between ligands and proteins. In drug discovery, it is also responsible for drug specificity, metabolization, and adsorption (Bhardwaj et al., 2020). To confirm the stability of all the docked complexes, the hydrogen bonds between 4ASD-Foretinib, 4ASD-mol22, and 4ASD-T01 were estimated in a solvent environment during MD simulations (Figures 8E, F, G). It was found that Foretinib formed an average of 2.01 hydrogen bonds and 3.23 bond pairs within 0.35 nm of the active pocket of 4ASD. Similarly, the designed molecule T01 was linked to 4ASD in the binding site via an average of 3.97 hydrogen bonds, while the average number of pairs within 0.35 nm was 4.15. However, for the 4ASD/molecule 22 complex, the average number of hydrogen bonds was 2.09, and the average number of pairs within 0.35 nm was 3.45. Notably, the H-bonding plot revealed that compound T01 was likely to interact more strongly with the binding pockets of 4ASD throughout the simulation compared with molecule 22 and Foretinib. The Hydrogen bond analysis emphasized the significant roles played by specific amino acid residues, in addition to catalytic residues, in the complexes of Foretinib, Molecule 22, and the developed compound T01 (Table 6). The distribution of hydrogen bond numbers further showed that the complex of designed compound T01 formed hydrogen bonds with affinities ranging from high to low, which is comparable to the distribution of hydrogen bonds in the complexes of Foretinib (Figures 8H).

**TABLE 6:** The hydrogen bond occupancy of amino acid residues throughout the simulation in various protein-ligand complexes.

Complex	H-bond occupancy of amino acid residues
Foretinib	Asp1046 (both donor and acceptor) 10.48%, Lys868 (donor) 39.62%, Arg1027 (both donor and acceptor) 23.25%, Tyr1059 (Donor) 0.60%, Glu885 (acceptor) 70.46%
Molecule 22	Lys868 (donor) 64.37%, Asn923 (donor) 0.20%, Arg1051 (donor) 7.29%, Asp1046 (acceptor) 57.09%
Designed molecule T01	Lys868 (donor) 65.17%, Asp1046 (donor and acceptor) 41.82%, Asp1028 (acceptor) 3.49%, His1026 (donor) 0.10%

### Binding free energy analysis

The MM-PBSA method was used to determine the binding free energy (ΔE) between the VEGFR-2-Foretinib, VEGFR-2-molecule 22, and VEGFR-2-T01 complexes using the MmPbStat.py script for whole trajectories (Chen et al., 2016; Gomari et al., 2023). The total nonpolar, polar, and non-bonded interaction energies (electrostatic interaction and Van der Waals) were calculated for each complex and are displayed in Table 7. Foretinib, mol22, and T01 all bind to VEGFR-2 with free energies of −48.084, −34.943, and −59.176 kJ/mol, respectively, proving the validity of the molecular dynamic simulation model used in this study. The non-polar solvation free energy (Enon polar), electrostatic energy (Eele), and van der Waals energy (Evdw) all contributed to the binding energy of the two systems, but the polar energy (E polar) was undesirable, demonstrating the significance of the intermolecular van der Waal contribution. This is consistent with the docking study and MD simulation interactions, where the large interaction of the ligand with the hydrophobic binding pocket was observed.

**TABLE 7:** MMPBSA calculations of binding free energy for all complexes.

Complex	Binding energy (kJ/mol)	SASA energy (kJ/mol)	Polar solvation energy (kJ/mol)	Electrostaticenergy (kJ/mol)	Van der Waals energy (kJ/mol)
4ASD/Foretinib	−48.084 +/- 49.845	−23.295 +/- 1.286	356.488 +/- 94.279	−187.730 +/- 84.457	−193.548 +/- 21.570
4ASD/Mol22	−34.943 +/- 37.198	−22.269 +/- 1.009	451.645 +/- 64.355	−141.591 +/- 39.791	−173.847 +/- 16.793
4ASD/T01	−59.176 +/- 40.252	−23.316 +/- 1.252	317.898 +/- 60.344	−290.472 +/- 48.945	−212.166 +/- 16.943

## Conclusion

To build the 3D-QSAR model, a series of triazolopyrazine derivatives against the breast cancer cell line MCF-7 were collected, optimized, and calculated. Statistically, both the CoMFA and CoMSIA models provide good results, with *R* ^2^ > 0.9 and Q^2^ > 0.5. External validation and the Y-randomization test were used to compare the predictive quality of the 3D-QSAR model. Thus, we used 3D-QSAR to design and predict the properties of 6 novel compounds. The results show that the expected activity and ADME/T curves for these molecules are quite strong. The molecular docking results show better binding affinity in the range of—8.9 to–10 kcal/mol, respectively, and strong binding to VEGFR-2 through several interactions. The MD simulation was used to study the stability of the conformations with the lowest binding value of each complex. 4ASD/T01 are stable based on RMSD, RMSF, Rg and SASA. During this research, the calculation of MM-GBSA confirmed the result of molecular docking by showing that the novel compound Pred T01 is more stable and has the lowest binding energy. Compared to the leading known breast cancer drug (Foretinib) the proposed six molecules exhibit enhanced binding and inhibitory activity against VEGFR-2, the major breast cancer receptor. This result provided the basis for the synthesis of novel triazolopyrazine analogs as improved drugs which expand the number of the new potential agents to face the rising resistant breast cancer. Chemical synthesis and further experimental validation to assess the proliferative activities of the designed molecule represents the main limitation of the present study.

## Data Availability

The original contributions presented in the study are included in the article/Supplementary Material, further inquiries can be directed to the corresponding authors.
