# Supplementary material for: Potent VEGFR-2 inhibitors for resistant breast cancer: a comprehensive 3D-QSAR, ADMET, molecular docking and MMPBSA calculation on triazolopyrazine derivatives
Source: Front Mol Biosci. 2023 Nov 22;10:1288652. doi: 10.3389/fmolb.2023.1288652 (PMC10702966; doi:10.3389/fmolb.2023.1288652)
Supplement: Supplementary file 1 [file DataSheet1.docx]

**Potent VEGFR-2 Inhibitors for Resistant Breast Cancer: A Comprehensive 3D-QSAR, ADMET, Molecular Docking and MMPBSA Calculation on Triazolopyrazine Derivatives**

Soukayna Baammi^1.2^, Achraf El Allali^2^*, Rachid Daoud^1^*

**^1^**Chemical and Biochemical Sciences-Green Processing Engineering, Mohammed VI Polytechnic University, Ben Guerir, Morocco

**^2^** Bioinformatics Laboratory, College of Computing, Mohammed VI Polytechnic University, Ben Guerir, Morocco

*Correspondance: Rachid Daoud (rachid.daoud@um6p.ma); Achraf El Allali (achraf.elallali@um6p.ma)

Table S1: Chemical structures and anti-cancer effects of triazole pyrazine derivatives. *** Represents the test set.**

| 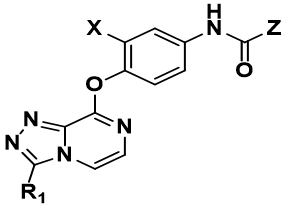 | | | | | |
| --- | --- | --- | --- | --- | --- |
| **Compounds** | **X** | **R1** | **Z** | **IC_50_ (μM)** | **pIC_50_** |
| 1 | H | H | 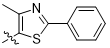 | 13.76 | 4.86 |
| 2 | H | H | 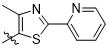 | 7.87 | 5.10 |
| 3 | H | H | 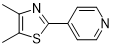 | 6.45 | 5.19 |
| 4 | H | H | 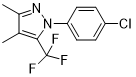 | 11.52 | 4.93 |
| 5* | H | H | 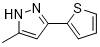 | 7.13 | 5.17 |
| 6* | F | H | 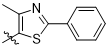 | 9.17 | 5.03 |
| 7 | F | H | 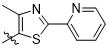 | 8.18 | 5.08 |
| 8 | F | H | 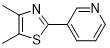 | 19.03 | 4.72 |
| 9* | F | H | 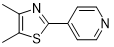 | 3.68 | 5.43 |
| 10 | F | H | 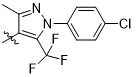 | 6.51 | 5.18 |
| 11 | F | H | 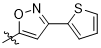 | 11.97 | 4.92 |
| 12 | H | CH_3_ | 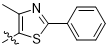 | 1.43 | 5.84 |
| 13 | H | CH_3_ | 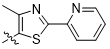 | 5.15 | 5.28 |
| 14 | H | CH_3_ | 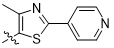 | 8.79 | 5.05 |
| 15 | H | CH_3_ | 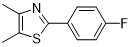 | 6.95 | 5.15 |
| 16 | H | CH_3_ | 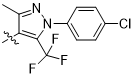 | 1.74 | 5.75 |
| 17 | H | CH_3_ | 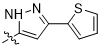 | 6.38 | 5.19 |
| 18 | F | CH_3_ | 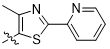 | 4.83 | 5.31 |
| 19 | F | CH_3_ | 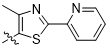 | 4.25 | 5.31 |
| 20 | F | CH_3_ | 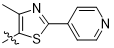 | 7.31 | 5.13 |
| 21* | F | CH_3_ | 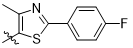 | 18.17 | 4.74 |
| **22** | **F** | **CH_3_** | 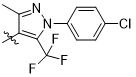 | **1.05** | **5.97** |
| 23 | F | CH_3_ | 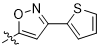 | 9.34 | 5.02 |

Table S2: pIC_50_ values observed and estimated and their residuals of different models for training and test sets. *Represents the test set.

| Comp. | pIC_50_ EXPER | CoMFA | | CoMSIA | |
| --- | --- | --- | --- | --- | --- |
|  |  | pIC_50_ pred | Residual | pIC_50_ PRED | Residual |
| 1 | 4,861 | 4,772 | 0,09 | 4,92 | -0,06 |
| 2 | 5,104 | 5,107 | 0,00 | 5,04 | 0,06 |
| 3 | 5,190 | 5,246 | -0,06 | 5,27 | -0,08 |
| 4 | 4,939 | 4,989 | -0,05 | 4,97 | -0,03 |
| 5* | **5,147** | **5,146** | 0,00 | **5,22** | -0,08 |
| 6* | **5,038** | **5,652** | -0,61 | **5,56** | -0,53 |
| 7 | 5,087 | 5,101 | -0,01 | 5,04 | 0,04 |
| 8 | 4,721 | 4,653 | 0,07 | 4,60 | 0,12 |
| **9*** | **5,434** | **5,112** | 0,32 | **4,99** | 0,44 |
| 10 | 5,186 | 5,294 | -0,11 | 5,35 | -0,16 |
| 11 | 4,922 | 4,799 | 0,12 | 4,80 | 0,12 |
| 12 | 5,845 | 5,721 | 0,12 | 5,67 | 0,17 |
| 13 | 5,288 | 5,279 | 0,01 | 5,33 | -0,04 |
| 14 | 5,056 | 5,127 | -0,07 | 5,08 | -0,02 |
| 15 | 5,158 | 5,194 | -0,04 | 5,24 | -0,08 |
| 16 | 5,759 | 5,745 | 0,01 | 5,75 | 0,01 |
| 17 | 5,195 | 5,23 | -0,03 | 5,26 | -0,07 |
| 18 | 5,316 | 5,311 | 0,01 | 5,26 | 0,06 |
| 19 | 5,372 | 5,53 | -0,16 | 5,51 | -0,14 |
| 20 | 5,136 | 5,118 | 0,02 | 5,11 | 0,03 |
| **21*** | **4,741** | **6,077** | -1,34 | **5,98** | -1,23 |
| 22 | 5,979 | 5,831 | 0,15 | 5,82 | 0,16 |
| 23 | 5,030 | 6,098 | -0,07 | 6,12 | -0,09 |

Table S3: CoMFA and CoMSIA models

| **Models** | R2 | **Q2** | SEE | F | NOC | Fractions | | | | |
| --- | --- | --- | --- | --- | --- | --- | --- | --- | --- | --- |
|  |  |  |  |  |  | Ster | Elec | Hyd | Don | Acc |
| **CoMFA** | **0.936** | **0.575** | **0.102** | **29.265** | **6** | **0.64** | **0.36** | - | - |  |
| S | 0.886 | 0.511 | 0.136 | 15.51 | 4 | 1 | - | - | - | - |
| E | 0.874 | -0.011 | 0.143 | 13.874 | 3 |  | 1 | - |  | - |
| /H | 0.881 | 0.238 | 0.139 | 14.862 | 2 | - | - | 1 | - |  |
| D | 0.804 | -2.011 | 0.173 | 10.861 | 4 | - | - | - | 1 | - |
| A | 0.814 | -1.011 | 0.133 | 11.423 | 3 |  | - |  | - | 1 |
| **SE** | **0.938** | **0.575** | **0.100** | **30.437** | **6** | **0.642** | **0.358** | **-** | **-** | **-** |
| SH | 0.792 | 0.24 | 0.132 | 15.51 | 4 | 0.414 | - | 0.586 | - | - |
| /SD | 0.810 | 0.314 | 0.165 | 13.874 | 6 | 0.567 | - | - | 0.433 | - |
| SA | 0.866 | 0.063 | 0.214 | 14.862 | 4 | 0.529 | - | - | - | 0.471 |
| /EH | 0.826 | -0.124 | 0.168 | 10.861 | 6 | - | 0.416 | 0.584 | - | - |
| /ED | 0.801 | 0.034 | 0.126 | 15.51 | 4 | - | 0.722 | - | 0.278 | - |
| /EA | 0.833 | -0.109 | 0.132 | 13.874 | 6 | - | 0.528 | - | - | 0.472 |
| HD | 0.825 | 0.041 | 0.165 | 14.862 | 4 | - | - | 0.696 | 0.304 | - |
| /HA | 0.867 | 0.363 | 0.187 | 10.861 | 6 | **-** | - | 0.625 | - | 0.375 |
| /DA | 0871 | -0.224 | 0.188 | 15.51 | 6 | - | - | - | 0.410 | 0.590 |
| /SEH | 0.830 | 0.517 | 0.152 | 13.874 | 6 | 0.441 | 0.436 | - | - | 0.123 |
| /SED | 0.807 | 0.094 | 0.125 | 14.862 | 4 | 344 | 0.358 | - | 0.298 | - |
| /SEA | 0.811 | 0.451 | 0.156 | 10.861 | 5 | 0.328 | 0.361 | - | - | 0.311 |
| /SHD | 0.837 | 0.412 | 0.661 | 11.423 | 3 | 0.320 | - | 0.378 | 0.302 | 0.320 |
| /SHA | 0.882 | 0.402 | 0.716 | 15.51 | 3 | 0.276 | - | 0.477 | - | 0.247 |
| /SDA | 0.816 | -0.061 | 0.118 | 13.874 | 4 | 0.389 | - | - | 0.325 | 0.286 |
| /EHD | 0.831 | 0.243 | 0.117 | 13.811 | 4 | - | 0.344 | 0.398 | 0.259 | - |
| /EDA | 0.845 | 0.343 | 0.113 | 12.921 | 3 | - | 0.324 | 0.403 | - | 0.273 |
| /EHA | 0.893 | 0.246 | 0.102 | 13.028 | 3 | - | 0.358 | 0.417 | - | 0.225 |
| /HDA | 0.881 | 0.434 | 0.115 | 12.726 | 3 | - | 0.441 | - | 0.295 | 0.263 |
| /SEHD | 0.853 | 0.420 | 0.099 | 11.001 | 3 | 0.236 | 0.258 | 0.263 | 0.243 | - |
| /SEHA | 0.792 | 0.345 | 0.098 | 12.223 | 3 | 0.191 | 0.302 | 0.326 | - | 0.181 |
| /SHDA | 0.790 | 0.24 | 0.086 | 12.094 | 4 | 0.250 | - | 0.301 | 0.258 | 0.191 |
| /SEDA | 0.891 | 0.413 | 0.114 | 13.912 | 3 | 0.270 | 0.292 | 0.253 | - | 0.185 |
| /EHDA | 0.878 | 0.201 | 0.117 | 12.061 | 3 | - | 0.275 | 0.303 | 0.236 | 0.186 |
| ESHDA | 0.844 | 0.063 | 0.104 | 14.183 | 3 | 0.197 | 0.224 | 0.225 | 0.212 | 0.142 |

Table S4: Y-randomization tests of CoMFA model.

| Model | R | R^2^ | Q^2^ |
| --- | --- | --- | --- |
| **Original** | **0,967471** | **0,936** | **0,575** |
| Random 1 | 0,273892 | 0,075017 | -0,17618 |
| Random 2 | 0,212572 | 0,045187 | -0,13993 |
| Random 3 | 0,153446 | 0,023546 | -0,17335 |
| Random 4 | 0,254428 | 0,064733 | -0,23148 |
| Random 5 | 0,146177 | 0,021368 | -0,48822 |
| Random 6 | 0,066002 | 0,004356 | -0,23614 |
| Random 7 | 0,320761 | 0,102888 | -0,11691 |
| Random 8 | 0,174289 | 0,030377 | -0,3285 |
| Random 9 | 0,328955 | 0,108211 | -0,05237 |
| Random 10 | 0,095043 | 0,009033 | -0,26328 |
| Random 11 | 0,356434 | 0,127045 | -0,12684 |
| Random 12 | 0,16607 | 0,027579 | -0,25201 |
| Random 13 | 0,186162 | 0,034656 | -0,19383 |
| Random 14 | 0,094228 | 0,008879 | -0,53512 |
| Random 15 | 0,258633 | 0,066891 | -0,11954 |
| Random 16 | 0,383946 | 0,147415 | -0,06156 |
| Random 17 | 0,071877 | 0,005166 | -0,17362 |
| Random 18 | 0,034263 | 0,001174 | -0,18332 |
| Random 19 | 0,384763 | 0,148043 | -0,21386 |
| Random 20 | 0,162983 | 0,026563 | -0,11109 |

Table S5: Y-randomization tests of CoMSIA model.

| Model | R | R^2^ | Q^2^ |
| --- | --- | --- | --- |
| Original | 0.938337 | 0.938 | 0.575 |
| Random 1 | 0.558322 | 0.311724 | 0.069291 |
| Random 2 | 0.351862 | 0.123807 | -1.179310 |
| Random 3 | 0.459327 | 0.210981 | -0.126375 |
| Random 4 | 0.396112 | 0.156904 | -0.104323 |
| Random 5 | 0.149328 | 0.022298 | -0.331680 |
| Random 6 | 0.125115 | 0.015653 | -0.344207 |
| Random 7 | 0.189267 | 0.035822 | -0.200889 |
| Random 8 | 0.395346 | 0.156298 | -0.272040 |
| Random 9 | 0.308552 | 0.095204 | -0.658444 |
| Random 10 | 0.186403 | 0.034746 | -0.366701 |
| Random 11 | 0.318325 | 0.101331 | -0.553358 |
| Random 12 | 0.277028 | 0.076744 | -0.178903 |
| Random 13 | 0.658223 | 0.433258 | 0.279803 |
| Random 14 | 0.5220019 | 0.272485 | 0.077946 |
| Random 15 | 0.361386 | 0.130600 | -0.581810 |
| Random 16 | 0.250431 | 0.062715 | -0.473001 |
| Random 17 | 0.177203 | 0.031401 | -0.479175 |
| Random 18 | 0.353609 | 0.125039 | -0.219135 |
| Random 19 | 0.401300 | 0.161042 | -0.193189 |
| Random 20 | 0.233923 | 0.054720 | -0.393374 |


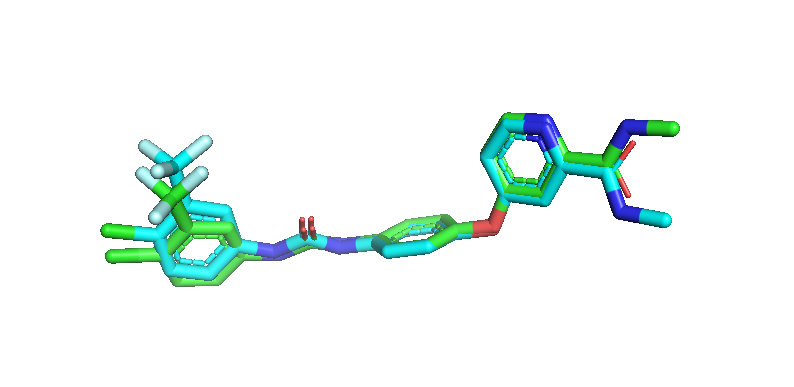


Figure S1: Re-docking pose with an RMSD value of 1.01 Å (Green = native ligand, Blue= docked ligand)
